# Supplementary material for: Taste cues elicit prolonged modulation of feeding behavior in Drosophila
Source: iScience. 2022 Sep 17;25(10):105159. doi: 10.1016/j.isci.2022.105159 (PMC9529979; doi:10.1016/j.isci.2022.105159)
Supplement: Document S1. Figures S1 and S2 [file mmc1.pdf]

iScience, Volume *25*

Supplemental information

Taste cues elicit prolonged modulation of feeding behavior in *Drosophila*

Julia U. Deere and Anita V. Devineni

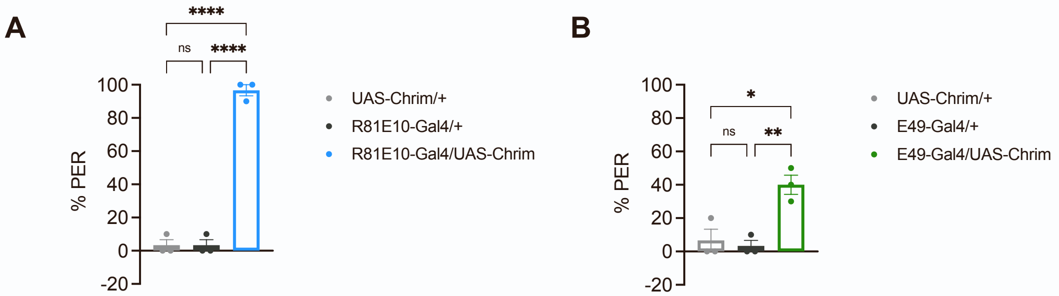

Figure S1, related to Figure 1. Activation of the Fdg neuron or MN9 motor neuron acutely elicits PER

(A-B) Percent of flies acutely showing PER during light activation of the Fdg neuron (A) or MN9 motor neuron (B) (n = 3 sets of flies). Genotypes were compared using one-way ANOVA followed by Tukey's multiple comparisons test.

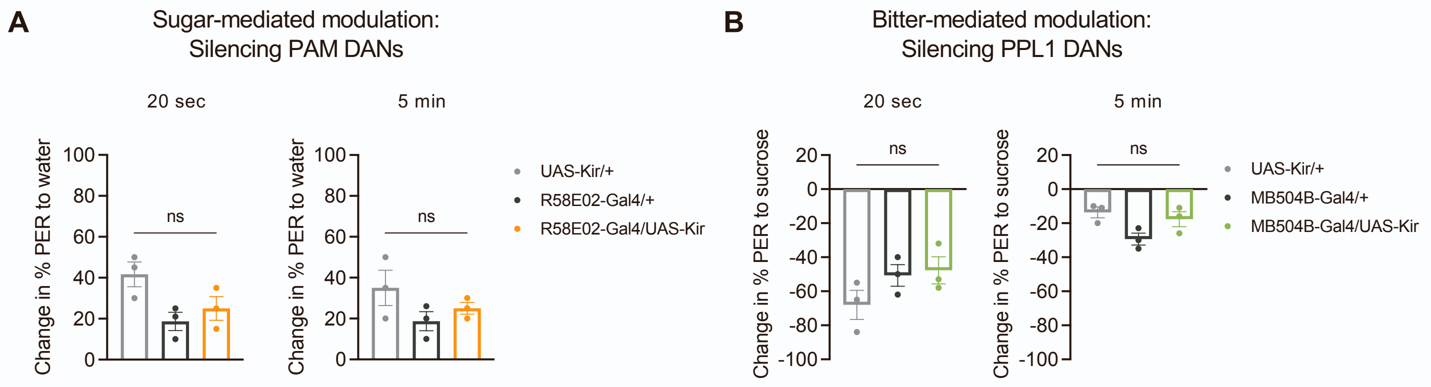

Figure S2, related to Figure 5. Chronic silencing of PAM or PPL1 DANs does not affect prolonged modulation of PER

(A) Effect of chronically silencing PAM DANs (*R58E02-Gal4* driving *UAS-Kir2.1*) on PER modulation by sugar. Graphs show change in PER to water tested 20 sec (left) or 5 min (right) after sugar presentation.

(B) Effect of chronically silencing PPL1 DANs (*MB504B* split-*Gal4* driving *UAS-Kir2.1*) on PER modulation by bitter taste. Graphs show change in PER to sucrose tested 20 sec (left) or 5 min (right) after quinine presentation.

In all panels, genotypes were compared using one-way ANOVA followed by Tukey’s multiple comparisons test.
